# An Eight-Year Clinic Experience with Clozapine Use in a Parkinson’s Disease Clinic Setting

**DOI:** 10.1371/journal.pone.0091545

**Published:** 2014-03-19

**Authors:** Nawaz Hack, Sarah M. Fayad, Erin H. Monari, Umer Akbar, Angela Hardwick, Ramon L. Rodriguez, Irene A. Malaty, Janet Romrell, Aparna A. Wagle. Shukla, Nikolaus McFarland, Herbert E. Ward, Michael S. Okun

**Affiliations:** 1 Department of Neurology, University of Florida Center for Movement Disorders and Neurorestoration, Gainesville, Florida, United States of America; 2 Department of Psychiatry, University of Florida, Gainesville, Florida, United States of America; University of Iowa Carver College of Medicine, United States of America

## Abstract

**Background:**

To examine our eight year clinic-based experience in a Parkinson’s disease expert clinical care center using clozapine as a treatment for refractory psychosis in Parkinson's disease (PD).

**Methods:**

The study was a retrospective chart review which covered eight years of clozapine registry use. Statistical T-tests, chi-square, correlations and regression analysis were used to analyze treatment response for potential associations of age, disease duration, and Hoehn & Yahr (H&Y) score, and degree of response to clozapine therapy.

**Results:**

There were 36 participants included in the analysis (32 PD, 4 parkinsonism-plus). The characteristics included 30.6% female, age 45–87 years (mean 68.3±10.15), disease duration of 17–240 months (mean 108.14±51.13) and H&Y score of 2 to 4 (mean 2.51±0.51). The overall retention rate on clozapine was 41% and the most common reasons for discontinuation were frequent blood testing (28%), nursing home (NH) placement (11%) and leucopenia (8%). Responses to clozapine across the cohort were: complete (33%), partial (33%), absent (16%), and unknown (16%). Age (r = −0.36, p<0.01) and H&Y score (r = −0.41, p<0.01) were shown to be related to response to clozapine therapy, but disease duration was not an associated factor (r = 0.21, p>0.05).

**Conclusions:**

This single-center experience highlights the challenges associated with clozapine therapy in PD psychosis. Frequent blood testing remains a significant barrier for clozapine, even in patients with therapeutic benefit. Surprisingly, all patients admitted to a NH discontinued clozapine due to logistical issues of administration and monitoring within that setting. Consideration of the barriers to clozapine therapy will be important to its use and to its continued success in an outpatient setting.

## Introduction

Parkinson’s disease (PD) is the second most common neurodegenerative disorder the United States [1]. Non-motor symptoms such as psychosis can lead to complications such as disability, and early nursing home placement [2]–[7]. Regulatory agencies such as the Food and Drug Administration (FDA) do not recommend a particular antipsychotic drug for the treatment of psychosis associated with PD [8], [9]. The FDA has also issued a black box warning concerning the increased incidence of morbidity and mortality that can occur with the use of atypical antipsychotics drugs [5], [10]–[12].

Among experts [9] managing PD, clozapine has frequently been recognized as the most efficacious drug for addressing PD related psychosis [2], [4], [13]–[15]. Though highly efficacious, clozapine is frequently under-utilized due to the requirement for weekly blood draws to monitor for the small potential of drug-induced agranulocytosis [14], [16].

While there have been many studies examining the use of clozapine in the PD population, it has scarcely been examined in a single movement disorders clinic setting without the stringent inclusion criteria inherent in clinical trials. We review and share our eight year experience on the use of clozapine in a large experienced movement disorders clinic registry. We also report on the indications for clozapine treatment, patient outcomes, and reasons for clozapine discontinuation.

## Methods

The University of Florida (UF) Center for Movement Disorders and Neurorestoration has a formal registry and clinical coordinator assigned to record and follow patients treated with clozapine. Approval was obtained from the University of Florida’s institutional review board for a retrospective chart review that covered eight years of clozapine registry use. Informed consent was not obtained since records/information could be anonymized and de-identified prior to analysis.

The protocol for enrollment into the clozapine registry included a diagnosis of PD or Parkinsonism, psychotic symptoms, such as delusions and/or hallucinations and a baseline complete blood count (CBC). An enrollee in the registry was required to have blood work performed every week for six months. If blood work remained stable over this period, the patient was monitored biweekly. Transient neutropenia (Absolute neutrophil count (ANC) <1500 mm^3^) was considered a reason for discontinuation of clozapine [17].

The starting dose for all patients was 12.5 mg nightly. If the effect was subtherapeutic in the first week, this dose was increased to 25 mg. The dosage was gradually increased by 12.5 mg to 25 mg each week, if tolerated, until symptoms of psychosis were eliminated or greatly reduced.

UF had 36 PD and Parkinsonism patients enrolled in the clozapine registry from June 2005 to June 2012. Records were reviewed in detail for baseline descriptive characteristics: age, gender, and diagnosis and disease duration (in months) (Table 1). Additional data was collected on Hoehn/Yahr stage (H&Y) and also on adjustment of PD medications prior to initiating clozapine. Response to clozapine was rated as 0 (none), 1 (partial), or 2 (complete). The definitions for the ratings were complete (full resolution of symptoms), partial (symptoms reduced, insight retained and benign hallucinations) and no response (symptoms worsened or did not improve).

**Table 1:** Baseline characteristics of our sample.

	Mean Age in Years (SD)	Gender	Mean Disease Duration in Months (SD)	Mean Hoehn & Yahr Score (On medication)(SD)	Symptoms Prior to Clozapine	Response to Clozapine	Reason for Stopping Clozapine (if applicable)
**Parkinson’s Disease** **(N = 32)**	67.66 (10.54)	28.1% female 71.9% male	114.16 (49.95)	2.53 (.52)	90.6% psychosis; 3.1% punding; 3.1% DAWS; 3.1% impulse;control disorder	15.6% none; 34.4% partial;37.5% complete;12.5% unknown	28.1% blood draw inconvenience; 9.4% died; 3.1% decreased white blood cell; 6.3% hypotension; 3.1% Worsened Psychosis; 3.1% no benefit; 46.3% unknown
**Parkinsonism (N = 4)**	73.75(3.30)	50% female 50% male	60.00 (35.33)	2.38 (.48)	75.0% psychosis	25.0% none; 25.0%partial; 50.0% unknown	25.0% blood draw inconvenience; 25.0% decreased white blood cell; 25.0% no benefit; 25.0% paralyzing feeling (side effect)
**Overall** **Sample** **(N = 36)**	68.33 (10.15)	30.6% female 69.4% male	108.14 (51.13)	2.51 (.51)	88.9% psychosis; 2.8% punding; 2.8% DAWS; 2.8% impulsecontrol disorder	16.7% none; 33.3% partial;33.3% complete;16.7% unknown	27.8% blood draw inconvenience; 5.6% died; 8.3% decreased white blood cell; 2.8% hypotension; 2.8% Delirium; 2.8% no benefit; 2.8% paralyzing feeling (side effect); 47.2% unknown

All charts were reviewed for data on neurological and physical symptoms, symptom resolution, length of treatment, dosages of clozapine, and if applicable, the reason for stopping clozapine. Participants that were treated with levodopa or dopamine agonists and who suffered psychosis were titrated to the lowest dose of levodopa and dopamine agonists without exacerbating motor symptoms prior to initiating clozapine. Levodopa was reduced to the lowest possible dose that alleviated the motor symptoms. Drugs that could potentially exacerbate psychosis such as amantadine and anticholinergics were discontinued if possible in an effort to alleviate the situation without the use of clozapine [18]–[26]. We did not exclude any patients with psychosis who possibly could benefit from clozapine therapy and we did not exclude those on cholinesterase medications.

### Statistical Methodology

T-tests, chi-square, correlations and regression analysis using SPSS Version 21 examined age, disease duration, and Hoehn and Yahr scores, and how they related to clozapine treatment response.

## Results

Participants (n = 36, see Table 1) were 30.6% female, ranged in age from 45–87 years (M = 68.33, SD = 10.15), had disease duration of 17–240 months (M = 108.14, SD = 51.13), and ranged in Hoehn & Yahr score from 2–4 (rated in half-digit intervals; M = 2.51 SD = . 51). Thirty-two had Parkinson’s disease and 4 had Parkinsonism. Those with Parkinson’s disease versus Parkinsonism did not significantly differ on age, disease duration, or Hoehn & Yahr score (Table 1), though t-tests may have been hindered by uneven sample sizes between groups.

Initial treatments for psychosis included a decrease in levodopa (n = 18, 56%), and/or trial with quetiapine (n = 19, 59%). Mean treatment duration was 18 months (range 1 week-72 months). Among the idiopathic Parkinson’s group (Table 1), 2/32 (6%) patients stopped clozapine due to decreased white blood cell count, 2/32 (6%) as a result of medication related side effects (hypotension and increased agitation), and 2/32 (6%) reported no benefit. Twenty eight percent terminated clozapine therapy citing personal preference including the inconvenience of frequent blood draws and lack of resources to have them performed weekly. Six of 32 (19%) reported benefit but clozapine was discontinued due to hospitalization (n = 1), nursing home placement (n = 3), or death (n = 2). All Parkinsonism patients discontinued clozapine. Reasons for discontinuation included decreased white blood cell count (n = 1), no benefit (n = 1), side effect (feeling of being paralyzed) (n = 1), and nursing home placement (n = 1). Eleven percent of our patients were placed in a nursing home and all discontinued clozapine therapy due to the nursing home’s reluctance to continue therapy despite the monitoring occurring by our center staff. Forty-one percent remained on clozapine and reported partial or complete relief from hallucinations.

**Figure 1:**
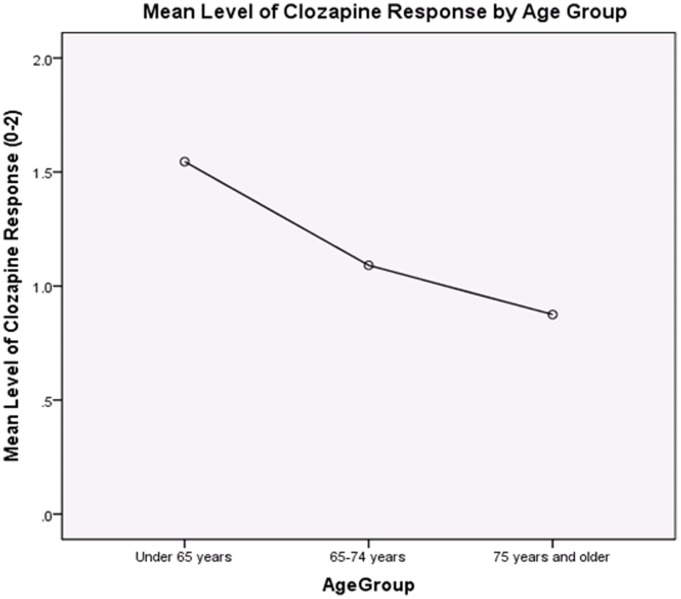
Relationship between response to clozapine use and age groups.

**Figure 2:**
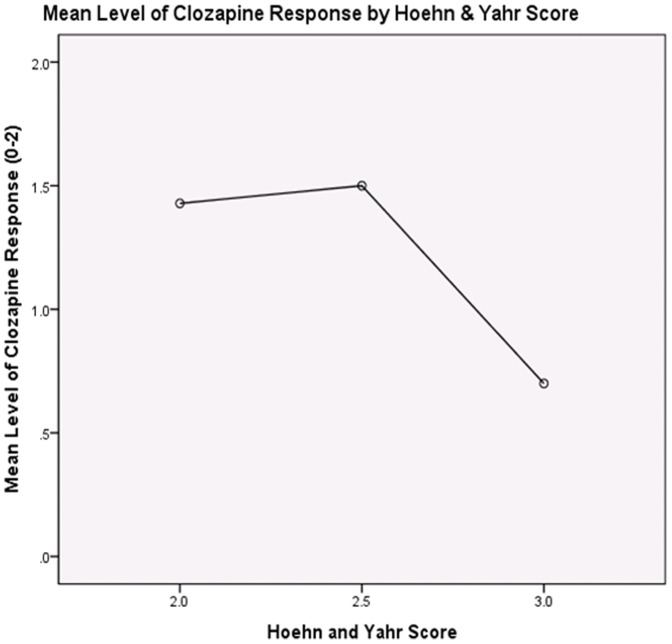
Relationship between responses to clozapine when correlated to H&Y scores.

As seen in Table 2, there was no association between subject age and disease duration (r = . 24, ns), or between disease duration and clozapine response (r = . 21, ns). There were significant relationships between age and Hoehn & Yahr (r = . 44, p<.01), and Hoehn & Yahr and disease duration (r = . 52, p<.01). Both age (r = −.36, p<. 01) and Hoehn & Yahr (r = −.41, p<.01) were significantly related to clozapine response, suggesting that patients who were older or had higher Hoehn & Yahr scores were less likely to respond to clozapine (Figures 1 and 2).

**Table 2:** Correlation between the continuous variables of age, disease duration, and H&Y score and clozapine use.

	Age in Years	Disease Duration in Months	Hoehn & Yahr Score	Response to Clozapine
**Age in Years**	1	.24	.44**	−.36*
**Disease Duration in Months**	.24	1	.52**	−.21
**Hoehn & Yahr Score**	.44**	.52**	1	−.41*

**Correlation is significant at p<.01, *correlation is significant at p<.05.

As shown in Table 3, Hoehn & Yahr score predicted clozapine response (β = −.41, p<.05) and accounted for 17% of the variance. Disease duration did not predict response (β = 11, ns) and explained only 1% of the variance. Age increased amount of variance explained to 21%, but removed the significant independent effect of Hoehn & Yahr.

**Table 3:** Response to Clozapine by Age, Disease Duration, and Hoehn & Yahr Score (regression analysis).

Model	Variables Included	Unstandardized Coefficient B(Std.Error)	Standardized Coefficient (β)	T	R Square
**1**	Constant	2.90 (.72)	–	4.03**	.17
	Hoehn & Yahr Score	−.70 (.29)	−.41	−2.40*	–
**2**	Constant	3.02 (.77)	–	3.91**	.18
	Hoehn & Yahr Score	−.82 (.39)	−.49	−2.11*	–
	Disease Duration	.00 (.00)	.11	.48	–
**3**	Constant	3.57 (.96)	–	3.72**	.21
	Hoehn & Yahr Score	−.63 (.44)	−.37	−1.45	–
	Disease Duration	.00 (.00)	.10	.41	–
	Age	−.01 (.02)	−.20	−.97	–

**p<.01, *p<.05.

## Discussion

In our study, 11% of the cohort required nursing home placement due to refractory psychosis and the consequent inability of caregivers to manage psychosis in the home setting [27], [28]. This nursing home admission resulted in an unfortunate discontinuation of clozapine therapy by the nursing home staff and physicians. Our center did offer to monitor each of these patients, and all were part of the UF registry. The reason for discontinuation was most frequently cited as a concern about the side effects, the FDA black box warning and the need for weekly blood monitoring and blood draws. We are uncertain as to why our registry had so many discontinuations in the nursing home setting, but we suspect that more education on the topic could positively influence nursing home policies. Additionally, since our center is a tertiary referral facility with most patients traveling long distances for evaluation, it is possible that a geographical bias led to the large number of nursing home discontinuations.

The retention rate in our movement disorders clinic cohort was 41%. This is notably less than many of the clinical trials examining the use of clozapine in patients with PD. It is important to keep in mind that twenty eight percent of our patients stopped clozapine due to the frequency of blood draws (Table 1). Many of these patients had complete resolution of their behavioral symptoms, yet frequent blood draws persisted as a barrier to continuing therapy. In clinical practice assessing the patient’s commitment and also providing education on the necessity of blood work required for safe therapy could be important to the overall success of clozapine for any individual patient. There has been a very limited literature on the barrier of blood draws in clozapine therapy, and not unexpectedly our study found it to be a major barrier [4], [6], [29].

It is important to appreciate that some patients in our study opted out of treatment before reaching therapeutic doses. It is difficult to evaluate this factor as many of our patients did not progress in titration and may not have achieved a therapeutic drug level.

In our cohort there was an 8.3% incidence of transient neutropenia, which resolved upon discontinuation of clozapine. No cases of agranulocytosis were seen (Table 1). The mortality rate over an eight-year follow up was 5.6%. The deaths in our cohort were not directly attributed to clozapine therapy or to complications of clozapine. Agranulocytosis (ANC<500 mm3) and transient neutropenia (ANC<1500 mm3) remain the feared complications of clozapine therapy. In previous literature, the incidence of transient neutropenia was reported as 3%, whereas the risk of agranulocytosis was 0.8% at one year and 0.9% after 1.5 years of therapy. Pons and colleagues reported neutropenia in 3% of 271 patients in a 5-year follow-up study, however, there were no cases reported of agranulocytosis [9].

Of note, our clinic cohort had higher H & Y disease staging scores and the cohort was in general one of advanced age. Although our sample size was small, our study revealed that clozapine, which is widely believed to be the best treatment for psychosis in PD, was not as effective in an older population and in later disease stages (Figures 1 and 2). This relationship between age and H&Y has been observed in other studies [30]–[35] and may reflect the presence of more refractory symptoms in advance Parkinson disease or alternatively reflect other co-morbidities. However, and in contrast to our study, some published clinical trials have shown a better response to clozapine in patients with similar age and H&Y stage (Table 4) [2]–[4], [9], [36]–[41]. We postulate based on our clinical experience that this important difference may have been due to barriers to the use of clozapine in a typical clinical population, such as geographical access to providers, and access to pharmacies capable of adhering to the stringent protocol of monitoring and dispensing medication [29], [42]. Also other factors may have included transportation issues, and sensitivity to the discomfort of long-term blood draws. Further, the stringent inclusion criteria employed in the published studies may impart a selection bias toward patients motivated and capable of complying for the entire duration of any given study.

**Table 4:** Comparison of clozapine studies in the literature.

Study Author/Year	Type of Study	Number of subjects	Drug or Placebo Comparison	Dosage of Clozapine	Positive Clinical Outcome(Yes/No)	Mean Age of Patients	Average H&YStaging Score	Outcome Scales Utilized
**Merims 2006 (1)**	Randomized, single blind,open label	27	Quetiapine	13.1+−7.0	Yes, but not different fromquetiapine using CGIC	71.8+−9 years	2.9+−0.3	CGIC
**Pintor 2012 (2)**	Randomized, single blind,open label	16	Ziprasidone	32.14+−12.19	No, when comparedto Ziprasidone	74.25+−9.57 (only in Clozapine group)	3.87+−0.99 (only in Clozapine group)	SAPS, BPRS
**Pollak 2004 (3)**	Randomized, double blind,placebo controlled trial	60	Placebo	35.8	Yes	71.2+−7.4	3.3+−0.9	CGI, PANSS
**Factor 2001 (4)**	Randomized, double blind,placebo controlled trial	53	Placebo	28.78	Yes	71.3+−8.2	2.7+−0.8	CGI, BPRS
**Morgante 2004 (5)**	Randomized, single blind,open label,	45	Quetiapine	26+−12	Yes, but equal to quetiapine	69+−10.7 (clozapinegroup only)	Not reported	CGI-S, BPRS
**Goetz 2000 (6)**	Randomized, double blind,open label	15	Olanzapine	Unknown	Yes	Not reported	Not reported	SAPS
**Ellis 2000 (7)**	Randomized, double blind,open label	10	Risperidone	62.5+−32.7	Yes, but not statisticallysignificant	74.0+−5.9 (clozapinegroup only)	Not reported	BPRS
**French Clozapine Parkinson** **Study Group 1999 (8)**	Randomized, double blind,placebo controlled trial	60	Placebo	36+−14	Yes	72+−8	3.2+−1.1	CGI, PANSS
**The Parkinson Study Group** **1999 (9)**	Randomized, double blind,placebo controlled trial	60	Placebo	24.7	Yes	70.8+−8.6	2.6+−0.9	CGI, BPRS
**Wolters 1990 (10)**	Randomized, double blindplacebo controlled trial	6	Placebo	170.8	Yes	69.8 (range 59 to 81)	III–IV	SAPS, BPRS

**Legend:** CGIC: Clinical Global Impression of Change.

SAPS: Scale for Assessment of Positive Symptoms.

BPRS: Brief Psychiatric Rating Scale.

PANSS: Positive and Negative Syndrome Scale.

Our clinic cohort has taught us the importance of factoring in nursing home placement and the patient commitment to continued blood draws as a major factors that can influence the commitment to clozapine therapy even in the setting of therapeutic benefit. We have reflected on this experience and have initiated better education of the nurse home facilities staff and physicians when patients are treated with clozapine in this setting.

This current study was limited by a small sample size and lack of validated scales to carefully measure psychosis. It should be noted that there are only a few validated scales that can be utilized for measuring PD psychosis [43]. In our study, the subjective rating scales used by clinicians were a weakness; however, they were applied consistently across the population. Despite the limitations, the study provides practical insight into the use of clozapine in the context of an expert movement disorders clinic setting. More data will be needed to determine the efficacy of clozapine in patients with increasing age and H&Y scores. Our long-term outcome was in general similar to previously reported clozapine trials especially in mortality [36], [44], [45]. Moreover, Factor et al 2001 had reported that 18 (34%) of the 53 patients enrolled in the extension arm of the PSYCLOPS (PSYchosis and CLOzapine in the treatment of Parkinsonism) trial were either hospitalized or died during the trial period and 10% of subjects had passed away at the time the trial was published [44]. Clozapine remains an attractive choice for the treatment of PD related psychotic symptoms as was shown in one of the largest retrospective studies published by Trosch et al 1998 [46], [45]. With proper surveillance and protocols for administration, agranulocytosis appears to be a relatively rare complication. Clinicians should be aware of the potential barriers to use in the outpatient clinic setting.
